# Nonlocal Means Two Dimensional Histogram-Based Image Segmentation via Minimizing Relative Entropy

**DOI:** 10.3390/e20110827

**Published:** 2018-10-28

**Authors:** Chundi Jiang, Wei Yang, Yu Guo, Fei Wu, Yinggan Tang

**Affiliations:** 1 College of Electrical and Information Engineering, Quzhou University, Quzhou 324000, China; 2 Logistic Engineering College, Shanghai Maritime University, Shanghai 200135, China; 3 State GRID Quzhou Power Supply Company, No.6, XinHe Road, Quzhou 324000, China; 4 Institute of Electrical Engineering, Yanshan University, Qinhuangdao 066004, China

**Keywords:** image segmentation, thresholding, non-local filter, two dimensional histogram

## Abstract

Spatial correlation information between pixels is considered to be very important in thresholding methods. However, it is often ignored and thus unsatisfied segmentation results maybe obtained. To overcome this shortcoming, we propose a new image segmentation approach by taking not only pixels’ spatial information but also pixels’s gray level into account. First, a non-local mean filter is imposed on the image. Then the filtered image and the original image together are adopted to build a two dimensional histogram, it is called non-local mean two dimensional histogram. Finally, a minimum relative entropy criteria is used to select the ideal thresholding vector. Since the non-local mean filter process is performed in a neighborhood of current pixel, it carries out the spatial information of current pixel. Segmentation results on several images illustrate the effectiveness of the proposed thresholding method, whose segmentation accuracy are greatly improved compared to most existing thresholding methods.

## 1. Introduction

In the area of computer vision, image segmentation is a primary pre-processing step. The primary goal of image segmentation is to partition the image into several regions. In each region, image characteristic such as brightness, color, and texture are similar to some extend, while between different regions, these characteristics are obviously different. Image segmentation techniques had widely been adopted by different practical application task such as cell segmentation [1], object detection in SAR image [2], defect detection [3,4], etc.

To deal with image segmentation, many approaches and strategies had been developed. For example, turbopixel/superpixel segmentation methods [5,6], watershed segmentation methods [7,8],active contour models [9,10], clustering based methods [11,12], deep learning-based methods [13,14], thresholding methods [15,16],and so on. The advantages and disadvantages of these methods are summarized in Table 1.

Thresholding is very popular because it is relatively simple and can be implemented more easy. Thresholding methods assume that the gray level histogram of an image has distinct peaks and valleys and therefore the objects could be distinguished from background via a threshold. Usually, an ideal threshold is determined by maximizing or minimizing an objective function constructed from gray level histogram to select an ideal threshold. For example, Otsu proposed to maximize between-class variance to select threshold value [17], Kittler proposed to minimize classification error to select threshold [18]. Pun first introduced entropy as an objective function for image thresholding segmentation [19], in which the posteriori entropy of the object and background was firstly calculated, and then the upper bound of them were maximized. After this, Wong et al. present an improved version of Pun’s approach by imposing some inequality constraints on posteriori entropy, which characterizes the regions’ uniformity and shape [20]. Kapur proposed another entropy based thresholding method called maximum entropy thresholding algorithm. The maximum entropy method calculated the entropy of objects and background, then the sum of them is maximized [21]. In [22], Li et al. suggested to minimize the difference between original image and segmented image to selected threshold. To achieve this, the concept of cross-entropy was used as the criteria.

The above classical thresholding methods use only gray information of images, while the spatial information between pixels is ignored. Therefore, they often produce some segmentation error. For instance, an identical threshold may not suitable to two different images with the same gray level histogram, and possibly cannot correctly segment the two images. To overcome this shortcoming, the spatial information between pixels should be taken into account in the segmentation process. To achieve this goal, Abutaleb proposed a novel concept, i.e., two dimensional histogram. Two dimensional histogram is a L×L matrix, each elements of it represents the occurrence probability of gray level pair (i,j), where *i* denotes the gray level of pixel in the original image and *j* the neighborhood smoothed image of original image [23]. Since the neighborhood smoothed image contains the spatial information among pixels, and thus, the spatial information was integrated into the selection of threshold. After Abutaleb’s work, several authors extend the classical thresholding methods to two dimensional thresholding methods by adopting two dimensional histogram. For example, two-dimensional Reny’s entropy thresholding method [24], two dimensional Otsu thresholding method [25] and two dimensional Tsallis entropy thresholding method [26]. Inspired by Abutaleb’s idea, many researchers constructed different two dimensional histograms using other spatial information between pixels. Xiao suggested to use similarity of a pixel with its neighborhood pixels as the spatial information to build two dimensional histogram, which is named as gray level spatial correlation (GLSC) histogram [27]. In [28], Adiljan adopted edge information and gray level of pixels to construct two dimensional histogram [28]. In Adiljan’s method, the gradient of original image was firstly computed, and then, the orientation histogram of the gradient image was calculated and used as edge information. The resulted two dimensional histogram is called 2D direction histogram. Also, Xiao presented another method to construct two dimensional histogram by combining original images’s gray level and gradient magnitude, the obtained histogram is called GLGM histogram [29].

As far as the two dimensional histograms mentioned above is concerned, the core idea is to apply a certain filter to the original image to obtain the spatial information between pixels. Abutaleb used a mean filter, which is restricted in a local neighborhood of a size 3 × 3. However, in some situation the local mean may lost fine details, for example, points, lines and edges, of an image. Observing these facts, a non-local mean filter [30] is adopted in this paper. Compared with local mean filter, non-local mean filter computes the weighted mean of all possible pixels in the image which is similar to the target pixel. The non-local filtered image and the original image together are used to constructed two dimensional histogram. The ideal two dimensional threshold vector is obtained by minimizing the relative entropy.

The rest of this paper is organized as follows. In Section 2, the non-local mean filter is briefly reviewed and then non-local mean two dimensional histogram is constructed. In Section 3, the threshold selection process by minimizing relative entropy is described. In Section 4, experimental results and discussion are presented in details. Lastly, the conclusion are conducted in Section 5.

## 2. Non-Local Mean Two Dimensional Histogram (NLMTDH)

### 2.1. Non-Local Mean Filter

Let X(i) denote the gray level of pixel *i* in image *I*. In non-local mean filter, the estimated value of pixel *i* is the weighted average of other pixels’s in image *I*, which is calculated as

(1) $$Y\left(i\right)=\sum_{j\in I}w\left(i,j\right)X\left(j\right),$$

where w(i,j) are the weights reflecting the similarity between pixel *i* and *j*, which is calculated as

(2) $$w\left(i,j\right)=\frac{1}{Z(i)}e^{-\frac{{∥X\left(N_{i}\right)-X\left(N_{j}\right)∥}^{2}}{h^{2}\sigma^{2}}},$$

where $N_{k}$ denotes a square neighborhood with a fixed size, whose center locates at pixel *k*, $X(N_{i})$ the gray level vector inside the square neighborhood $N_{i}$. σ is the standard deviation of the Gaussian kernel, and *h* represent the filtering degree. Z(i) is a normalizing constant as

(3) $$Z\left(i\right)=\sum_{j}e^{-\frac{{∥X\left(N_{i}\right)-X\left(N_{j}\right)∥}^{2}}{h^{2}\sigma^{2}}}.$$

The non-local means compares the grey level in a geometrical configuration in a whole neighborhood as well as in a single point.

### 2.2. Construction of NLMTDH

Let *J* be the non-local filtered image of original image *I*. The size of the two images is M×N, their gray level belongs to set {0,1,⋯,L−1}. I(x,y) and J(x,y) be the gray level of the pixel at (x,y), where x=1,2,…,M and y=1,2,…,N. Let $n_{ij}$ be the total number of pixels such that I(x,y)=i and J(x,y)=j, NLMTDH is defined as

(4) $$p_{ij}=\frac{n_{ij}}{M\times N}.$$

**Figure 1:**
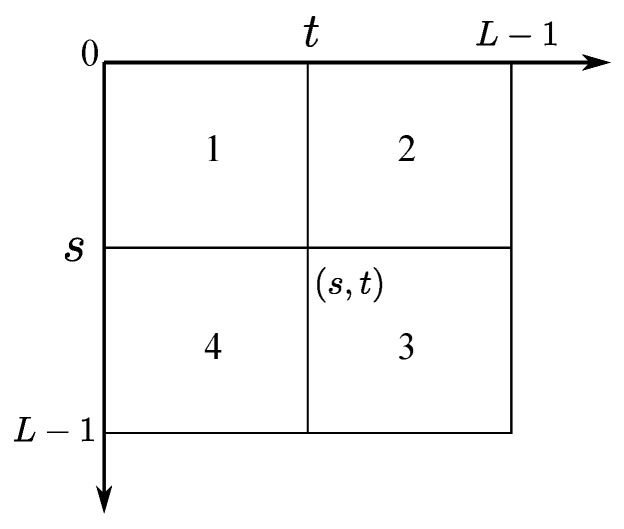
Non-local means two dimensional histogram.

NLMTDH $P=\{p_{ij};i,j=0,1,\cdots ,L-1\}$ is a L×L matrix, and is shown in Figure 1.

Assume a two dimensional (2D) threshold vector (s,t) divides NLMTDH into four regions, where *s* represents the threshold of original image and *t* the non-local means filtered image. Since the pixels in the interior of objects or background are similar each other, therefore, region 1 and 3 contain the information of objects and background, respectively. Region 2 and 4 contain the information of edges and noise.

## 3. Image Thresholding Based on NLMTDH Using Relative Entropy

### 3.1. Relative Entropy

Relative entropy, also called Kullback–Leibler (KL) divergence, is a measure to reflect the difference between two probability distribution *P* and *Q*. Let $P=\{p_{1},p_{2},\cdots ,p_{n}\}$ and $Q=\{q_{1},q_{2},\cdots ,q_{n}\}$ and satisfy $\sum_{i=1}^{n}p_{i}=\sum_{i=1}^{n}q_{i}=1$. The relative entropy between *P* and *Q* is defined as

(5) $$D\left(P,Q\right)=\sum_{i=1}^{n}p_{i}\log\frac{p_{i}}{q_{i}}.$$

Li and Lee [22] adopted relative entropy for thresholding selection. In image thresholding, *P* represents the original image distribution and *Q* the segmented image.

### 3.2. Threshold Selection Based on NLMTDH Using Relative Entropy

Consider the cast that there are only two classes in the image, let $C_{0}$ represent the background and $C_{1}$ object. As stated before, in Figure 1, region 1 and 3 contain the information of background and object, respectively. Let $P_{0}$ and $P_{1}$ be the occurrence probability of object and background at threshold vector (s,t). They are computed as

(6) $$P_{0}\left(s,t\right)=\sum_{i=0}^{s}\sum_{j=0}^{t}p_{ij},$$

and

(7) $$P_{1}\left(s,t\right)=\sum_{i=s+1}^{L-1}\sum_{j=t+1}^{L-1}p_{ij}.$$

The mean vector of the two classes are

(8) $$\mu_{0}=\left(\mu_{0i},\mu_{0j}\right)=\left(\frac{\sum_{i=0}^{s}\sum_{j=0}^{t}ip_{ij}}{P_{0}\left(s,t\right)},\frac{\sum_{i=0}^{s}\sum_{j=0}^{t}jp_{ij}}{P_{0}\left(s,t\right)}\right)$$

and

(9) $$\mu_{1}=\left(\mu_{1i},\mu_{1j}\right)=\left(\frac{\sum_{i=s+1}^{L-1}\sum_{j=t+1}^{L-1}ip_{ij}}{P_{0}\left(s,t\right)},\frac{\sum_{i=s+1}^{L-1}\sum_{j=t+1}^{L-1}jp_{ij}}{P_{0}\left(s,t\right)}\right),$$

respectively. Similar to [22], the relative entropy between the original image and the segmented image in NLMTDH at the threshold vector (s,t) is defined as

(10) $$\begin{array}{cc} D(P,Q|,s,t) & =\sum_{i=0}^{s}\sum_{j=0}^{t}\left(ip_{ij}\log\frac{i}{\mu_{0i}}+jp_{ij}\log\frac{j}{\mu_{0j}}\right)\\ & +\sum_{i=s+1}^{L-1}\sum_{j=t+1}^{L-1}\left(ip_{ij}\log\frac{i}{\mu_{1i}}+jp_{ij}\log\frac{j}{\mu_{1j}}\right). \end{array}$$

More details about how Equation (10) is defined, one can see Appendix A. Substituting Equations (8) and (9) into Equation (10) and after some manipulations, one can get

(11) $$\begin{array}{cc} D(P,Q|,s,t)= & M-P_{0}\left(s,t\right)\left(\mu_{0i}\log\mu_{0i}+\mu_{0j}\log\mu_{0j}\right)\\ & -P_{1}\left(s,t\right)\left(\mu_{1i}\log\mu_{1i}+\mu_{1j}\log\mu_{1j}\right), \end{array}$$

where $M=\sum_{i=0}^{L-1}\sum_{j=0}^{L-1}\left(ip_{ij}\log i+jp_{ij}\log p_{j}\right)$, which is a constant for the entire image. An ideal threshold vector $\left(s^{*},t^{*}\right)$ should be one that minimizes D(P,Q|,s,t), i.e.,

(12) $$\left(s^{*},t^{*}\right)=\arg\min D\left(P,Q|,s,t\right).$$

## 4. Experimental Results and Discussion

### 4.1. Results

To demonstrate the effectiveness of the proposed method, several real images are used to test the algorithm. The proposed method is compared with other methods including Otsu method, Kapur method, Minimum cross entropy (MCE) method, 2D histogram-based minimum cross entropy (2DMCE) method. These methods are implemented on an Intel Core(TM) i5-4200U 2.3GB platform with 8GB RAM using Matlab. The test images include *Ant*, *Bacteria*, *Block*, *geometric*, *Junk*, *Mask*, and two casting images.

In this paper, the misclassification error (ME) is adopted as objective criteria to evaluate the performance of the referenced methods. ME [31] is defined as

(13) $$\mathrm{ME}=1-\frac{|B_{o}\cap B_{T}\left|+\right|F_{o}\cap F_{T}|}{|B_{o}\left|+\right|F_{o}|},$$

for two classes segmentation, where $B_{o}$ and $F_{o}$ represent the background and foreground pixel set of ground-truth image, while $B_{T}$ and $F_{T}$ are the corresponding parts in the thresholded images, |.| represents the element number of a set. The value of ME lies in [0,1], where 0 implies a perfect segmentation and 1 for a completely wrong segmentation. A smaller ME value indicates a better segmentation quality.

**Figure 2:**
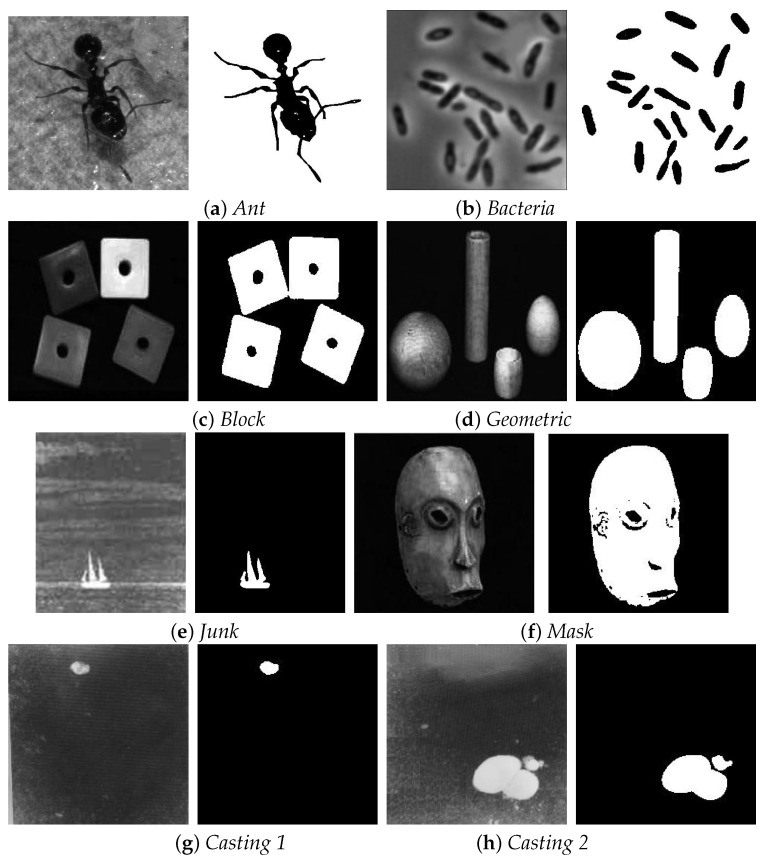
The testing images and their ground-truth images.

**Figure 3:**
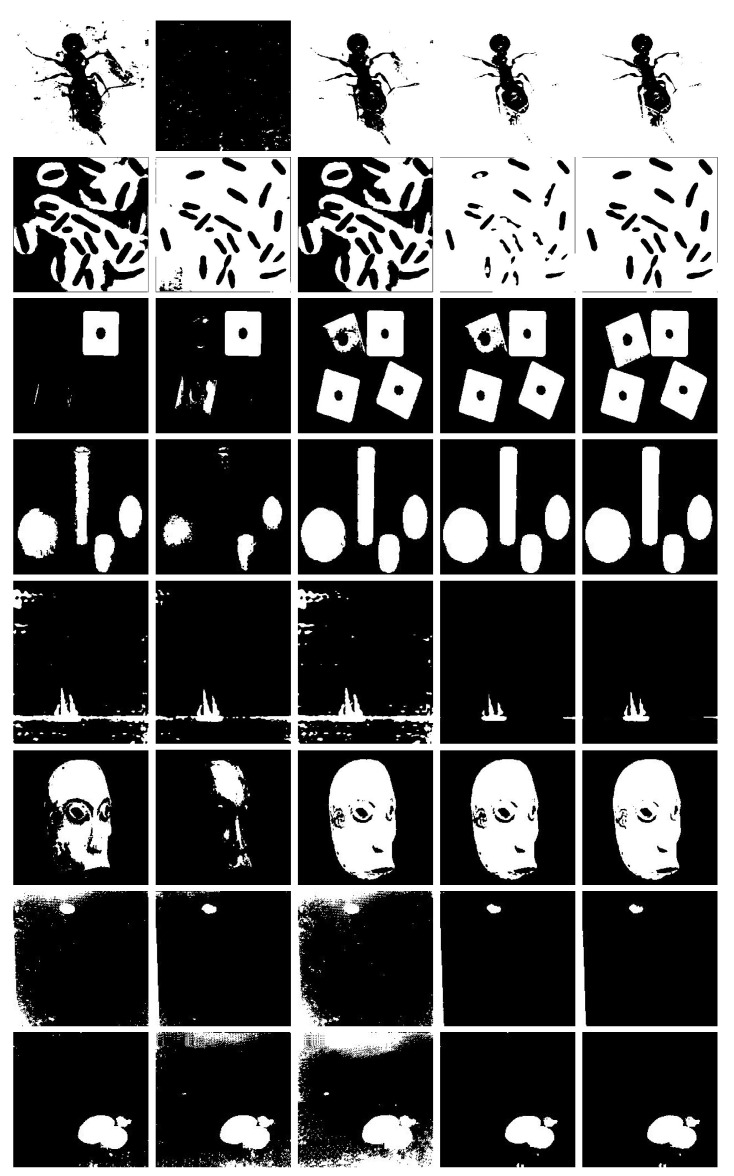
Thresholding results of test image using different methods. From left to right, the results are obtained by Otsu, Kapur, MCE,2DMCE and the proposed method.

Figure 2 shows all the testing images and corresponding ground-truth images, and Figure 3 exhibits the binary segmentation results through the referenced methods. As is shown in Figure 3, for the *Ant* image, the proposed method produces the best segmentation result, while Kapur’s method fails to give correct segmentation result. Otsu and MCE methods result in over-segmentation in some region and under-segmentation in other regions. 2DMCE method has some under-segmentation in the *Leg* part. For *Bacteria* image, Otsu and MCE method produce incorrect segmentation result. In the segmentation result of Kapur method, there are many background pixels are classified into foreground pixels. As for 2DMCE, there exits under-segmentation phenomenon. Our proposed method produces the best segmentation result with less segmentation error. For *Block* image, Otsu and Kapur method cannot fully extract the objects from background, while MCE and 2DMCE method has some segmentation error. Only our proposed method gives the correct segmentation result. For *geometric* image, the Otsu and Kapur method cannot separate the object from background correctly. The other three methods give satisfactory segmentation results. As far as *Junk* is considered, the Otsu, Kapur and MCE method can extract the object but there are much noises in the segmentation results. 2DMCE and our proposed method give satisfactory segmentation results, while our method gives more accurate result. As for *Mask* image, Otsu and Kapur method give poor segmentation results. MCE, 2DMCE and our method obtains acceptable segmentation results. For the two casting images, it can be seen that our method produces the best result.

Table 2 lists the obtained thresholds or threshold vectors for two dimensional histogram-based segmentation method and the ME performance index for different referenced methods. It is obvious that the ME of our proposed method is the smallest, indicating the best segmentation results are obtained by our method.

Table 3 lists the execution time of every method. It can be seen that one histogram-based thresholding methods including MCE, Otsu and Kapur expend less time than two dimensional histogram-based thresholding methods. The reason lies in two aspects. One is that it should filter the original image and then formulate two dimensional histogram. The other is that the threshold search range of two dimensional histogram is L×L, while *L* for one dimensional histogram. The larger the search range, the more time it needs.

### 4.2. Discussion

Constructing two dimensional histogram by combining the original image and its filtered version is a popular strategy to integrate the spatial information between pixels into the thresholding process, which had been proven to result in higher segmentation performance than a one dimensional histogram. Abutaleb’s method was the first and a successful try [23]. In [23], local mean filter was adopted. From theoretic viewpoint, local mean filter belongs to Gaussian filter, which can smooth edges and details of image. Since Abutaleb’s method assumed that the pixels inside objects or background are similar, while the pixels located at edge or border are different from those in objects or background. If local mean filter smoothed the edges, it is possible that some pixels at edge or noise will be classified into background or objects if Abutaleb’s method is used, and thus higher segmentation error maybe occur. Research results in [30] showed that non-local mean filter is superior to local mean filter. Non-local mean filter can preserve more edges and details than local mean filter in that it finds pixels that are similar to the current pixel in the entire image instead of a local neighborhood and then use the weighted mean of these pixels as the filtered value of current pixel. Therefore, our method can enhance the performance of Abutaleb’s method by reducing segmentation error. This enhancement has been demonstrated by experimental results. Of course, since non-local mean filter is not so effective for pepper and salt noise, if an image is corrupted by pepper and salt noise, its performance improvement will be limited. This is the limitation of our proposed method. In the future, one can develop combined filters to filter the original image if it is corrupted by complex noise. This will be our future effort.

Our proposed method belongs to thresholding method. Usually, its performance is not as good as other sophisticated methods, such as CNN-based method [13]. The main reason is that it uses less information of the image.

## 5. Conclusions

In this paper, a new thresholding method is proposed based on non-local mean two dimensional histogram. First, the proposed method adopts non-local mean filter to filter the original image. This process can incorporate spatial information between pixels into the filtered image. Then, a non-local mean two dimensional histogram is constructed according the original image and the filtered image. Finally, the minimum relative entropy of the objects and background is calculated based on non-local mean two dimensional histogram, and the optimal threshold vector is determined by minimizing the relative entropy of the objects and background. In experiments, the proposed method is used to segment several real images and compared to some existing thresholding methods. It is shown that the proposed method can obtain better segmentation performance.

## Figures and Tables

**Table 1:** Comparison of several segmentation methods.

Methods	Advantages	Disadvantages
superpixel [5,6]	reduce redundant information; less complexity	cannot locate the edges accurately
watershed [7,8]	simple and intuition	usually result in over segmention
active contour models [9,10]	rigorous mathematical base;	sensitive noise; high computation complexity
clustering [11,12]	intensive value is enough; simple	the number of cluster cannot be determined automatically; spatial information is ignored;
deep learning [13,14]	high segmentation accuracy	large computation burden
thresholding [15,16]	simple, easy to be implemented	ignore spatial information

**Table 2:** The threshold and ME of different methods.

Image		The Proposed	2DMCE	MCE	OTSU	KAPUR
ant	threshold	52 52	44 45	69	84	183
	ME	0.0344	0.0379	0.0481	0.0829	0.8852
bacteria	threshold	65 65	44 45	98	99	70
	ME	0.0101	0.0605	0.4266	0.4398	0.0221
block	threshold	26 26	36 36	38	120	88
	ME	0.0183	0.0621	0.0679	0.2861	0.2613
geometric	threshold	36 36	36 36	41	70	126
	ME	0.0324	0.0347	0.0381	0.0986	0.2323
junk	threshold	187 186	209 206	129	134	158
	ME	0.0072	0.0090	0.0633	0.0492	0.0166
mask	threshold	23 24	28 30	30	57	116
	ME	0.0016	0.0129	0.0136	0.1134	0.2860
casting13	threshold	144 144	134 127	74	80	114
	ME	0.0115	0.0128	0.1046	0.0795	0.0170
casting18	threshold	154 153	158 154	92	138	114
	ME	0.0050	0.0063	0.2200	0.0074	0.0611

**Table 3:** The computation time of every method (second).

Image	The Propsed	2DMCE	MCE	Otsu	Kapur
ant	170.3123	21.1812	0.0317	0.0032	0.0067
bacteria	163.7921	23.9648	0.0097	0.0031	0.0081
block	94.4108	21.3898	0.0079	0.0027	0.0077
casting13	56.5179	9.2832	0.0066	0.0023	0.0043
casting14	56.6691	10.2639	0.0068	0.0027	0.0051
geometric	62.0219	18.1187	0.0073	0.0020	0.0059
junk	116.4295	14.0563	0.0061	0.0019	0.0045
mask	99.1112	22.3298	0.0088	0.0023	0.0057

## Appendix A. Derivation of Equation (10)

Let *I* be an image with *N* pixels, its gray level lies in $F=\{f_{1},f_{2},\cdots ,f_{N}\}$. Suppose a threshold *t* separates the image into two classes, i.e., object and background. The gray level of the segmented image is $G=\{g_{1},g_{2},\cdots ,g_{N}\}$. The mean gray level of the two parts are

(A1) $$\mu_{1}\left(t\right)=\frac{\sum_{f_{i}<t}^{}f_{i}}{N_{1}},\;\mu_{2}\left(t\right)=\frac{\sum_{f_{i}>t}^{}f_{i}}{N_{2}},$$

respectively, where $N_{1}$ and $N_{2}$ are the number of pixels that $f_{i}<t$ and $f_{i}\geq t$, respectively. In [22], the relative entropy between the original image and the segmented image is defined as

(A2) $$D\left(F,G|t\right)=\sum_{f_{i}<t}f_{i}\log\left(\frac{f_{i}}{\mu_{1}\left(t\right)}\right)+\sum_{f_{i}\geq t}f_{i}\log\left(\frac{f_{i}}{\mu_{2}\left(t\right)}\right).$$

By using the concept of histogram, Equation (A2) can be formulated in another way. Let $\left\{h_{1},h_{2},\cdots ,h_{L}\right\}$ be the histogram of the original image. Each elements $h_{j}$ represents the occurrence number or possibility of gray level *j* in the image. Using the concept of histogram, Equation (A2) can be equivalently formulated as

(A3) $$D\left(F,G|t\right)=\sum_{j=1}^{t-1}jh_{j}\log\left(\frac{j}{\mu_{1}\left(t\right)}\right)+\sum_{j=t}^{L}jh_{j}\log\left(\frac{j}{\mu_{2}\left(t\right)}\right),$$

where the mean $\mu_{1}\left(t\right)$ and $\mu_{2}\left(t\right)$ can be calculated using histogram as

(A4) $$\mu_{1}\left(t\right)=\frac{\sum_{j=1}^{t-1}jh_{j}}{\sum_{j=1}^{t-1}h_{j}},\;\mu_{2}\left(t\right)=\frac{\sum_{j=t}^{L}jh_{j}}{\sum_{j=t}^{L}h_{j}}.$$

By extending the definition of relative entropy-based on one dimensional histogram to two dimensional case, one can get Equation (10), i.e.,

(A5) $$\begin{array}{cc} D(P,Q|,s,t) & =\sum_{i=0}^{s}\sum_{j=0}^{t}\left(ip_{ij}\log\frac{i}{\mu_{0i}}+jp_{ij}\log\frac{j}{\mu_{0j}}\right)\\ & +\sum_{i=s+1}^{L-1}\sum_{j=t+1}^{L-1}\left(ip_{ij}\log\frac{i}{\mu_{1i}}+jp_{ij}\log\frac{j}{\mu_{1j}}\right). \end{array}$$

In Equation (A5), the term $ip_{ij}$ is similar to the term $jh_{j}$ in (A3), which represents the product of gray level *j* and its occurrence number.

## References

[1] Cao F., Cai M., Chu J., Zhao J., Zhou Z. (2017). A novel segmentation algorithm for nucleus in white blood cells based on low-rank representation. Neural Comput. Appl..

[2] Gao G. (2011). A parzen-window-kernel-based CFAR algorithm for ship detection in SAR Images. IEEE Geosci. Remote Sens. Lett..

[3] Malarvel M., Sethumadhavan G., Bhagi P.C.R., Kar S., Thangavel S. (2017). An improved version of Otsu’s method for segmentation of weld defects on X-radiography images. Opt. Int. J. Light Electron Opt..

[4] Yuan X.C., Wu L.S., Peng Q. (2015). An improved Otsu method using the weighted object variance for defect detection. Appl. Surf. Sci..

[5] Stutz D., Hermans A., Leibe B. (2017). Superpixels: An Evaluation of the State-of-the-Art. Comput. Vis. Image Underst..

[6] Levinshtein A., Stere A., Kutulakos K.N., Fleet D.J., Dickinson S.J., Siddiqi K. (2009). TurboPixels: Fast superpixels using geometric flows. IEEE Trans. Pattern Anal. Mach. Intell..

[7] Ciecholewski M. (2017). River channel segmentation in polarimetric SAR images: Watershed transform combined with average contrast maximisation. Expert Syst. Appl..

[8] Cousty J., Bertrand G., Najman L., Couprie M. (2010). Watershed cuts: Thinnings, shortest path forests, and topological watersheds. IEEE Trans. Pattern Anal. Mach. Intell..

[9] Ding K., Xiao L., Weng G. (2017). Active contours driven by region-scalable fitting and optimized Laplacian of Gaussian energy for image segmentation. Signal Process..

[10] Balla-Arabé S., Gao X. (2014). Geometric active curve for selective entropy optimization. Neurocomputing.

[11] Qureshi M.N., Ahamad M.V. (2018). An improved method for image segmentation using K-means clustering with neutrosophic logic. Procedia Comput. Sci..

[12] Parida P., Bhoi N. (2018). Fuzzy clustering based transition region extraction for image segmentation. Eng. Sci. Technol. Int. J..

[13] Zhao X., Wu Y., Song G., Li Z., Zhang Y., Fan Y. (2018). A deep learning model integrating FCNNs and CRFs for brain tumor segmentation. Med. Image Anal..

[14] Milletari F., Ahmadi S.A., Kroll C., Plate A., Rozanski V., Maiostre J., Levin J., Dietrich O., Ertl-Wagner B., Bötzel K. (2017). Hough-CNN: Deep learning for segmentation of deep brain regions in MRI and ultrasound. Comput. Vis. Image Underst..

[15] Khairuzzaman A.K.M., Chaudhury S. (2017). Multilevel thresholding using grey wolf optimizer for image segmentation. Expert Syst. Appl..

[16] Wang B., Chen L., Cheng J. (2018). New result on maximum entropy threshold image segmentation based on P system. Optik.

[17] Otsu N. (1979). A threshold selection method from gray-level histogram. IEEE Trans. Syst. Man Cybern..

[18] Kittler J., Illingworth J. (1986). Minimum error thresholding. Pattern Recognit..

[19] Pun T. (1980). A new method for gray-level picture thresholding using the entropy of the histogram. Comput. Vis. Graph. Image Process..

[20] Wong K.C., Sahoo P.K. (1989). A gray-level threshold selection method based on maximum entropy principle. IEEE Trans. Syst. Man Cybern..

[21] Kapur J.N., Sahoo P.K., Wong A.K.C. (1985). A new method for gray-level picture thresholding using the entropy of the histogram. Comput. Vis. Graph. Image Process..

[22] Li C.H., Lee C.K. (1993). Minimum cross entropy thresholding. Pattern Recognit..

[23] Abutaleb A. (1989). Automatic thresholding of gray-level pictures using two-dimensional entropy. Comput. Vis. Graph. Image Process..

[24] Sahoo P.K., Arora G. (2004). A thrsholding methd based on two-dimensional Renyi’s entropy. Pattern Recognit..

[25] Liu J.Z., Li W.Q. (1993). The automatic threshold of gray 2 level pictures via two 2 dimensional otsu method. Autom. Sin..

[26] Sahoo P.K., Arora G. (2006). Image thresholding using two-dimensional Tsallis-Havrda-Charvat entropy. Pattern Recognit. Lett..

[27] Xiao Y., Cao Z., Zhang T. Entropic thresholding based on gray-level spatial correlation histogram. Proceedings of the 19th International Conference on Pattern Recognition.

[28] Yimit A., Hagihara Y., Miyoshi T., Hagihara Y. (2013). 2-D direction histogram based entropic thresholding. Neurocomputing.

[29] Xiao Y., Cao Z., Yuan J. (2014). Entropic image thresholding based on GLGM histogram. Pattern Recognit. Lett..

[30] Buades A., Coll B., Morel J.M. A non-local algorithm for image denoising. Proceedings of the 2005 IEEE Computer Society Conference on Computer Vision and Pattern Recognition (CVPR’05).

[31] Li Z., Liu C., Liu G., Cheng Y., Yang X., Zhao C. (2010). A novel statistical image thresholding method. Int. J. Electron. Commun..

